# Whole Genome Sequence of *Marinobacter salarius* Strain SMR5, Shown to Promote Growth in its Diatom Host

**DOI:** 10.7150/jgen.39039

**Published:** 2019-10-01

**Authors:** Mats Töpel, Matthew I.M. Pinder, Oskar N. Johansson, Olga Kourtchenko, Anna Godhe, Adrian K. Clarke

**Affiliations:** 1 Department of Marine Sciences, University of Gothenburg, Göteborg, Sweden;; 2 Gothenburg Global Biodiversity Centre, Göteborg, Sweden;; 3 Department of Biological and Environmental Sciences, University of Gothenburg, Göteborg, Sweden.

**Keywords:** Whole Genome Sequencing, *Marinobacter*, Diatom, *Skeletonema*, Microbiome, Marine sediment

## Abstract

Attempts to obtain axenic cultures of the marine diatom *Skeletonema marinoi* often result in poor growth, indicating the importance of the microbiome to the growth of its host. In order to identify the precise roles played by these associated bacteria, individual strains were isolated, cultured and sequenced. We report the genome of one such strain - SMR5, isolated from a culture of *S. marinoi* strain R05AC sampled from top layer sediments of the Swedish west coast. Its genome of 4,630,160 bp consists of a circular chromosome and one circular plasmid, and 4,263 CDSs were inferred in the annotation. Comparison of 16S rRNA sequences and other markers, along with phylotaxonomic analysis, leads us to place strain SMR5 in the taxon *Marinobacter salarius*. Pathway analysis and previous experimental work suggest that this strain may produce a growth factor, as well as improve iron availability for its host via siderophores.

## Introduction

We have identified several bacterial strains living in association with strain R05AC of the chain-forming centric diatom *Skeletonema marinoi* 1-4. Bacteria and diatoms are known to interact in a number of ways, ranging from mutually beneficial nutrient exchange to parasitism 5, but the precise roles of the bacteria within the strain R05AC holobiont are as yet unknown. Therefore, we undertook a systematic approach to investigating these potential interactions by isolating individual bacterial strains associated with cultures of the diatom, then sequencing and annotating their genomes. One bacterial strain isolated this way was SMR5, whose genome is presented here.

The host culture, *S. marinoi* strain R05AC, was originally established from a germinated resting cell, collected in May 2010 from top layer sediment at 14m depth in Öresund, Sweden (55°59.16′ N, 12°44.02′ E). Strain SMR5 was isolated by dilution streaking from strain R05AC, and thenceforth maintained on marine agar plates (BD Difco - Fisher Scientific, USA). A distinguishing feature of strain SMR5, compared to the other bacteria identified so far in the *S. marinoi* microbiome, is the formation of white colonies with diffuse edges when grown on marine agar plates, compared to the firm colonies produced by all other bacterial species identified. Under microscopic observation, strain SMR5 can be seen to have a rod-shaped cell morphology and flagella.

Sequencing of strain SMR5 was performed using one SMRT cell on the PacBio RSII platform (Pacific Biosciences, Menlo Park, CA, USA), producing 163,482 unfiltered reads totalling 2.3 Gbp. The reads were filtered using the default parameters of SMRT Portal version 2.3.0's P_Filter module (minSubReadLength 500, readScore 0.80, minLength 100) 6, giving 101,723 filtered reads of 1.3 Gbp total. These reads were then assembled using the *de novo* assembler Canu version 1.3 7 (genomeSize parameter 4.5m). As Canu generates overlapping regions at the ends of contigs when assembling circular sequences, BLASTn 8 was used to identify the extent of these overlaps, in order to allow the contigs to be circularized. In this way, regions of 17,082 bp and 21,124 bp, highly similar between the beginning and end of their respective contigs, were manually trimmed from the start of the assembled chromosome and plasmid, respectively. Contig circularization was then confirmed by joining each contig's corresponding ends and realigning the reads using the RS_Resequencing.1 protocol on SMRT Portal version 2.3.0 (Pacific Biosciences, 6), which showed consistent read coverage across the joins. Correction of the assembled contigs was also performed during this step using the Quiver algorithm 6. The final assembly contained one circular chromosome and one circular plasmid, totalling 4,630,160 bp, with an average read coverage of 213.58x. The strain SMR5 chromosome is 4,386,892 bp long with a G+C content of 57.2%, and plasmid pSMR5 is 243,268 bp (G+C 53.7%) (assembly details summarized in Table 1). Annotation of the genome was performed using Prokka version 1.12beta 9, which inferred 4,263 CDSs (3,508 with functional predictions), 7 pseudogenes, 51 tRNAs, 9 rRNAs, 11 ncRNAs, and one tmRNA (annotation details summarized in Table 1).

**Figure 1:**
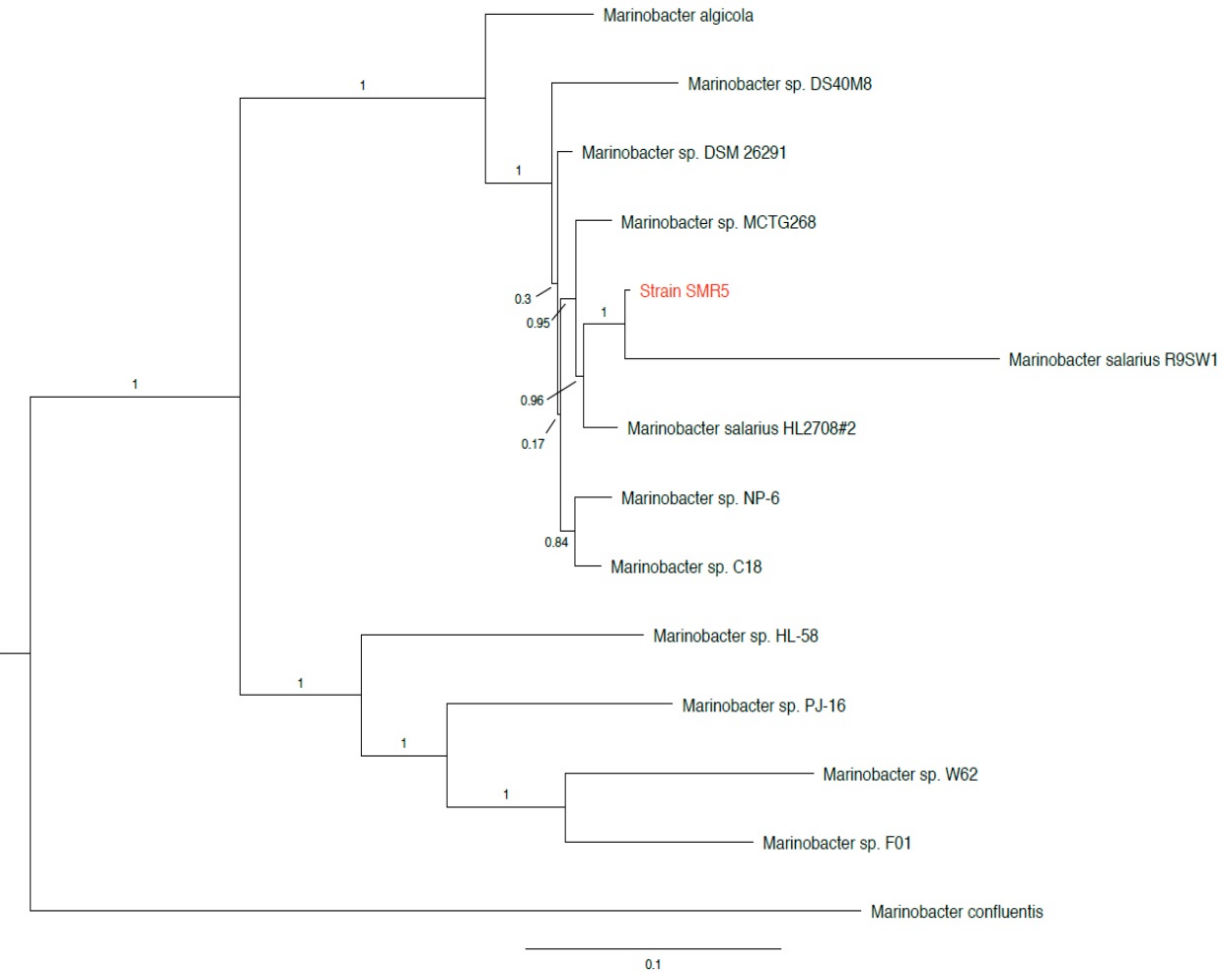
Clade of a phylogenetic tree showing placement of strain SMR5 (highlighted in red) within the family *Alteromonadaceae*. Adapted from tree generated with PhyloPhlAn version 0.99 11. Branch labels represent bootstrap values; scale bar indicates the mean number of nucleotide substitutions per site. Tree visualised with FigTree version 1.4.3 20.

The three 16S rRNA sequences of strain SMR5 (one differing from the other two by a single base substitution) share 99.8%/99.9% identity with the 16S sequence of *Marinobacter algicola* strain DG893^T^ (accession no. NZ_ABCP00000000), and 99.4%/99.5% identity with that of *Marinobacter salarius* strain R9SW1^T^ (accession no. NZ_CP007152). While not a type strain, the three identical 16S sequences of *M. salarius* strain HL2708#2 (accession no. NZ_CP021333) were found to have 99.9%/100% identity to strain SMR5. In addition, we found multiple sequences from unnamed *Marinobacter* sp. strains with ≥99.8% 16S identity to strain SMR5 in NCBI's RefSeq database 10. We then used PhyloPhlAn version 0.99 11 to perform a phylotaxonomic comparison of strain SMR5 against representatives of all whole-genome sequenced *Alteromonadaceae* species available on NCBI's RefSeq ftp site (ftp://ftp.ncbi.nlm.nih.gov/genomes/refseq/bacteria/; accessed 27 May 2019). This analysis showed that strain SMR5 is sister to *M. salarius* strain R9SW1^T^ (100% bootstrap support), with *M. algicola* appearing more distantly related (Figure 1).

Considering this apparent discrepancy between the 16S and phylotaxonomic analyses with regard to type strains, we also examined several conserved genes which have previously been used in *Marinobacter* classification - *gyrB*, *rpoB* and *rpoD* 12,13 - in order to more precisely classify strain SMR5. Whereas *M. algicola* strain DG893^T^ had <96% sequence identity to the homologs in strain SMR5, *M. salarius* strain R9SW1^T^ showed sequence identity of >99%. On this basis, we place strain SMR5 in the taxon *Marinobacter salarius*.

In terms of strain SMR5's relationship with its host, enhanced growth of *S. marinoi* strain R05AC in the presence of excess strain SMR5 has been observed in culture 14. Based on examination of the annotation using Pathway Tools version 21.0 15, our current hypothesis is that this may be caused by the production of indole-3-acetic acid (auxin) by strain SMR5. This hormone is known to promote growth in diatoms as well as plants 16, and the Pathway Tools analysis highlighted a nitrilase gene in the SMR5 genome (MARSALSMR5_03867) as being potentially involved in an auxin biosynthesis pathway (indole-3-acetate biosynthesis V in MetaCyc (https://metacyc.org; 17). Of additional note is the presence of a membrane transport protein (MARSALSMR5_00349) that, when compared to other *Marinobacter* proteins using BLASTp 8, appears to be an auxin efflux carrier, a protein present in many *Marinobacter* species. If this growth enhancement is indeed caused by auxin, it would be consistent with observations of other diatom-bacteria interactions, such as those between *Pseudo-nitzschia multiseries* and *Sulfitobacter* 16.

In addition, some phytoplankton-associated *Marinobacter* species are known to produce siderophores, which make iron available to both the bacteria and their phytoplankton host 18. Secondary metabolite gene cluster prediction using antiSMASH version 5.0.0 19 highlighted a region of the chromosome potentially involved in siderophore biosynthesis (corresponding to loci MARSALSMR5_03200 - MARSALSMR5_03239), indicating that strain SMR5 may also be capable of this.

## Nucleotide sequence accession numbers

This whole-genome project has been deposited in GenBank under the accession numbers CP020931 and CP020932 as part of BioProject no. PRJNA380207.

## Figures and Tables

**Table 1:** Statistics for the assembly and annotation of *Marinobacter salarius* strain SMR5.

	Total assembly	Chromosome	pSMR5
**Assembly**			
Number of reads (filtered)	101,723		
Number of bases (filtered)	1,318,128,196 bp		
Overlapping bases trimmed from start of contig		17,082 bp	21,124 bp
Final assembly size	4,630,160 bp	4,386,892 bp	243,268 bp
G+C content	57.0%	57.2%	53.7%
Average read coverage	213.58x		
**Annotation**			
CDS	4,263	3,999	264
Pseudogenes	7	6	1
tRNA	51	51	0
rRNA	9	9	0
ncRNA	11	10	1
tmRNA	1	1	0

## Abbreviations

CDS: coding sequence
SMRT: single-molecule real-time
ncRNA: non-coding RNA
tmRNA: transfer-messenger RNA
